# Structures and electrical properties of single nanoparticle junctions assembled using LaC_2_-encapsulating carbon nanocapsules

**DOI:** 10.1038/srep29708

**Published:** 2016-07-14

**Authors:** Manabu Tezura, Tokushi Kizuka

**Affiliations:** 1 Division of Materials Science, Faculty of Pure and Applied Sciences, University of Tsukuba, Tsukuba, Ibaraki 305-8573, Japan

## Abstract

As the miniaturization of integrated circuits advances, electronics using single molecules and nanosize particles are being studied increasingly. Single nanoparticle junctions (SNPJs) consist of two electrodes sandwiching a single nanoparticle. Nanocarbons with nanospaces in their center, such as fullerenes, carbon nanotubes, and carbon nanocapsules (CNCs), are expected to be elements of advanced SNPJs. In this study, SNPJs were assembled using lanthanum dicarbide (LaC_2_)-encapsulating CNCs and two gold (Au) electrodes by a nanotip operation inside a high-resolution transmission electron microscope. The atomic configuration and electrical resistance of the SNPJs were investigated *in situ*. The results implied that the electrical resistance of the SNPJ depended on the interface structures of the contacts between the CNC and Au electrodes, i.e., the contact electrical resistance, and the greatest portion of the current through the SNPJ flowed along the outermost carbon layer of the CNC. Thus, the resistance of the SNPJs using the CNCs was demonstrated and the electrical conduction mechanism of one of the CNC was discussed in this study.

Carbon nanocapsules (CNCs) are hollow carbon-layered shells that are occasionally referred to as giant fullerene molecules^1^,^2^,^3^,^4^. Various pure metals and carbides are encapsulated in the nanospaces^5^,^6^,^7^,^8^,^9^,^10^,^11^,^12^,^13^. The first studies of CNCs encapsulating nanoparticles addressed the encapsulation of lanthanum dicarbide (LaC_2_)^5^,^6^. Such hollow and nanoparticle-encapsulating CNCs are expected to be major elements of single nanoparticle junctions (SNPJs)^14^,^15^,^16^,^17^,^18^,^19^,^20^,^21^,^22^,^23^,^24^,^25^,^26^,^27^,^28^. The electrical properties of SNPJs are affected by the encapsulated particles and the structures of the nanoparticle and contact interfaces between the nanoparticles and electrodes. However, the structures of each component and interface in SNPJs have not been observed; therefore, the relationships between the structures and electrical properties are unclear. In particular, although the resistance and its origin of one isolated nanoparticle, including CNCs, are essential to designing SNPJs, they remain an open question because the structure and resistance of the contact interfaces between the nanoparticles and electrodes have not been estimated. *In situ* transmission electron microscopy (TEM) combined with nanotip operation techniques allows such simultaneous observations of structures and electrical properties^29^. In this study, we investigated the relationships between the structures and electrical properties of SNPJs assembled using LaC_2_-encapsulating CNCs.

## Results

### Au/LaC_2_-encapsulating CNC/Au

**Figure 1:**
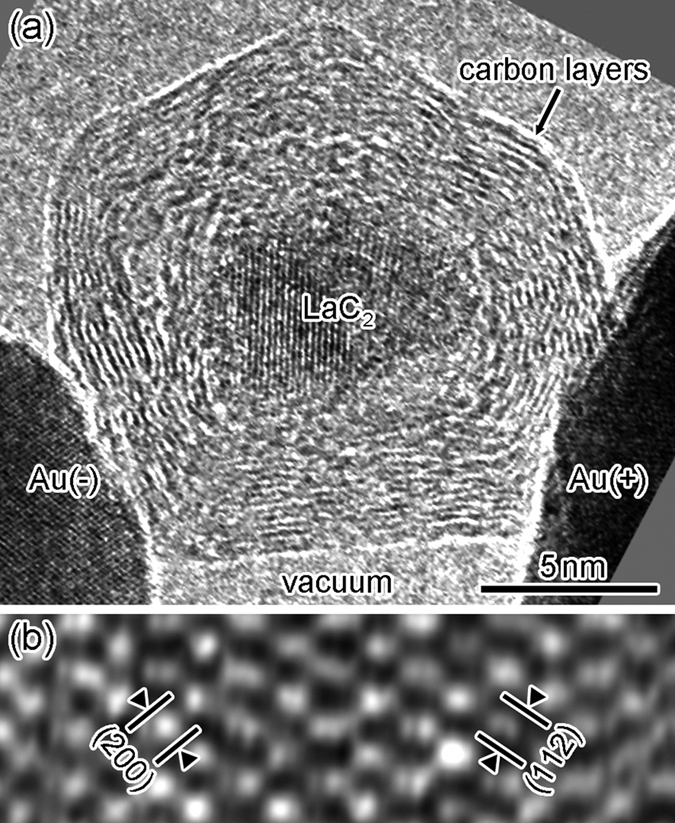
(**a**) High-resolution TEM image of a Au/LaC_2_-encapsulating CNC/Au SNPJ; (**b**) enlarged image of the LaC_2_ particle encapsulated in the CNC center. The two Au electrodes in the lower left and right regions shown in (**a**) are biased positively and negatively, respectively.

**Figure 2:**
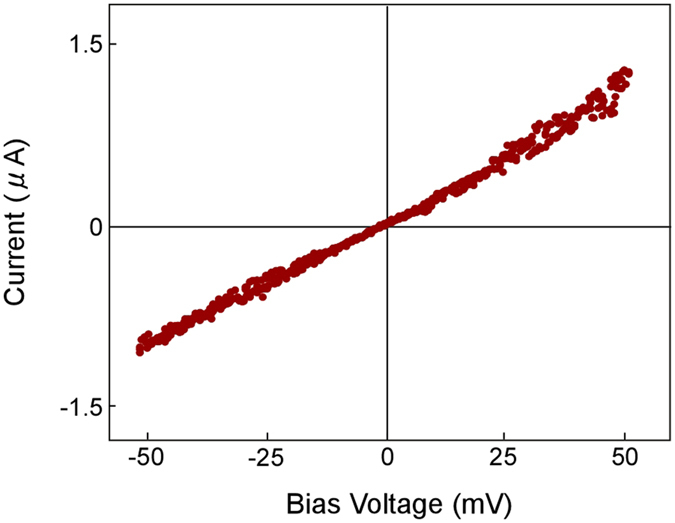
Current–voltage characteristics of the SNPJ presented in Fig. 1(a). The resistance near 0 V is 4.2 kΩ.

Figure 1(a) shows a high-resolution TEM image of an SNPJ assembled using a LaC_2_-encapsulating CNC and two gold (Au) electrodes (Supplementary Movie 1). The lower left and right dark regions are two Au electrodes biased positively and negatively, respectively. The regions around the CNC and Au electrodes correspond to vacuum. The diameters along the major and minor axes of the CNC are 18 and 12 nm, respectively. The CNC consists of 12 carbon layers. Figure 1(b) shows an enlarged image of the particle encapsulated in the center of the CNC shown in Fig. 1(a). The spacings of the lattice fringes observed in Fig. 1(b) are 0.20 and 0.18 nm, which correspond to the (112) and (200) planes of LaC_2_ with a tetragonal structure, respectively. Figure 2 shows the current–voltage characteristics of the SNPJ shown in Fig. 1(a), with fixed contact interfaces between the CNC and Au electrodes. The current–voltage curve of the SNPJ is linear at bias voltages from −50 to +50 mV. The resistance near 0 V is 4.2 kΩ.

We also measured the current of other SNPJs, in which the size of the CNCs and the contact areas between the CNC and the positive and negative electrodes were similar to those shown in Fig. 1, at bias voltages up to 500 mV. The current–voltage curve was linear at this voltage range with an almost constant differential conductance; the result was similar to that shown in Fig. 2. When higher bias voltage was applied, the encapsulated LaC_2_ nanoparticle moved outside the carbon shells. Therefore, the voltage was found to be critical for structural stability and electrical resistance measurements of the SNPJs.

**Figure 3:**
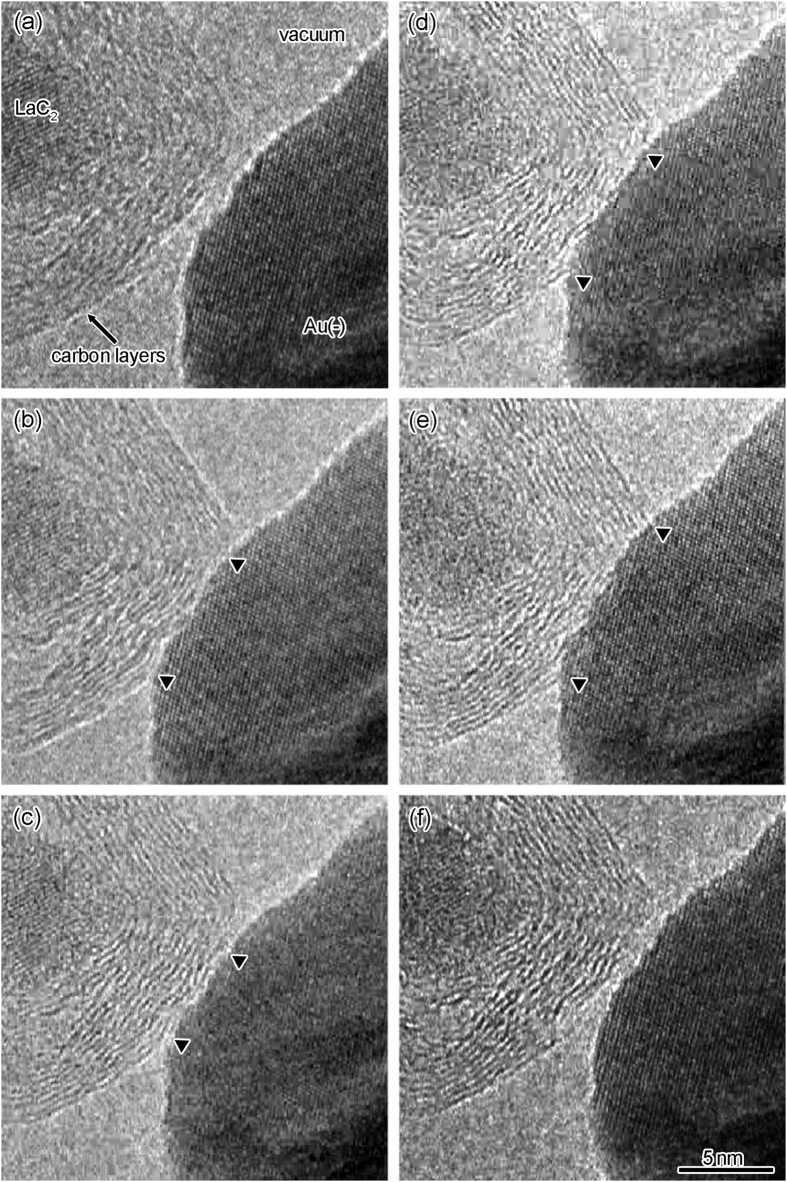
Time-sequence series of high-resolution TEM images of the contact region at the SNPJ shown in Fig. 1(a) during the contact, retraction, compression, and separation process between the CNC and the negative Au electrode: approaching the Au electrode (**a**); contact and retraction (**b,c**); compression (**c–e**); and separation (**f**). Both edges of the contact region between the carbon layers and Au electrode are indicated by two arrowheads, i.e., the contact region is located between the two arrowheads, as shown in (**b–e**).

**Figure 4:**
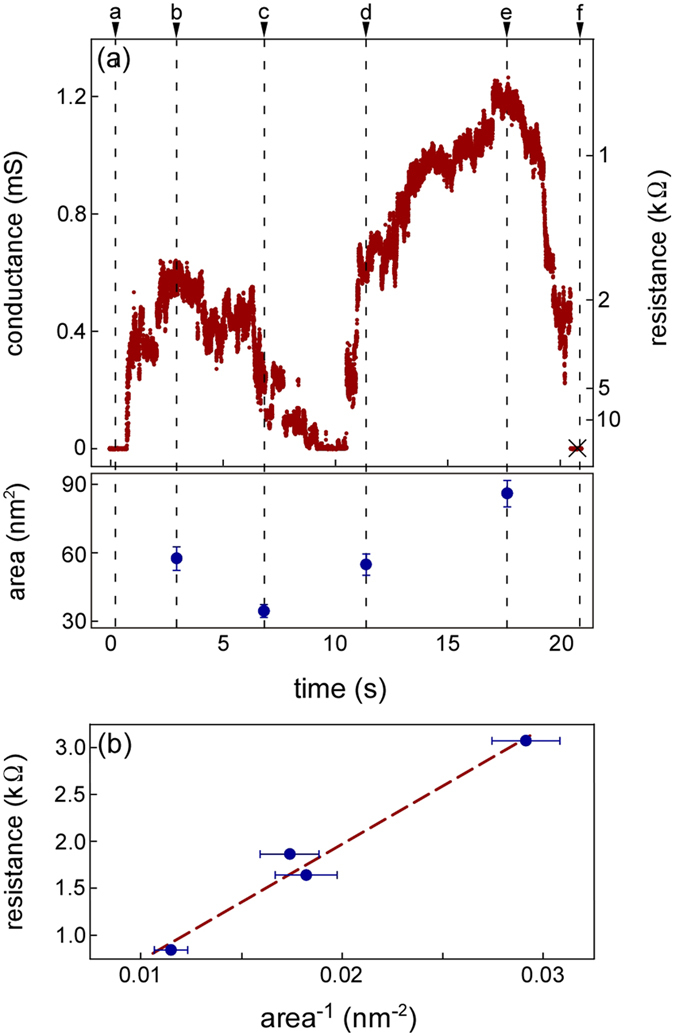
(**a**) Variations in electrical conductance (resistance) and contact areas during the process presented in Fig. 3 as a function of time. (**b**) Variations in electrical resistance as a function of the inverse of the contact areas observed in Fig. 3(b–e). The applied bias voltage was 47 mV. The times indicated by a–f in (**a**) correspond to the recording times of the images in Fig. 3(a–f), respectively. The cross in (**a**) indicates fracture. The dashed line in (**b**) is a linear approximation.

Figure 3 shows a time-sequence series of high-resolution images during the contact, retraction, compression, and separation process between the LaC_2_-encapsulating CNC and the negative Au electrode at the SNPJ shown in Fig. 1(a). In this process, the contact region between the CNC and the positive Au electrode was fixed. The applied bias voltage was fixed at 47 mV. The size of the contact region between the CNC and the negative Au electrode was controlled by manipulating the positive Au electrode. The electrical conductance (resistance) of the SNPJ and the contact areas during the process shown in Fig. 3 are illustrated in Fig. 4(a). To calculate the contact area, we assumed the shape of the contact area to be circular; the area was calculated from the diameter measured by the observed interface width. The times in Fig. 4(a–f) correspond to the observed times in Fig. 3(a–f), respectively. First, the CNC was allowed to approach the Au electrode (Fig. 4(a), time a), followed by contact and retraction (Fig. 3(b–c)) and compression (Fig. 3(c–e)). When the contact area became 54 nm^2^ (Fig. 3(b)), the electrical conductance increased to 0.5 mS (Fig. 4(a), time b). Then, the electrical conductance decreased as the CNC was pulled back from the Au electrode. When the contact area decreased to 36 nm^2^ (Fig. 3(c)), the electrical conductance decreased to 0.3 mS (Fig. 4(a), time c). Subsequently, the electrical conductance increased (Fig. 4, time c–e) as the contact area increased again (Fig. 3(c–e)). Finally, when the contact area increased to 91 nm^2^ (Fig. 3(e)), the electrical conductance increased to 1.2 mS (Fig. 4(a), time e). The electrical conductance (resistance) of the SNPJ shown in Fig. 1(a) was changed by the contact area of the interface between the CNC and the Au electrode. Since the electrical resistance depends on the inverse of the contact area, we plot the electrical resistance against the inverse of the areas in Fig. 4(b). A linear relationship was found between both parameters.

### Electrical resistance of capsule part

**Figure 5:**
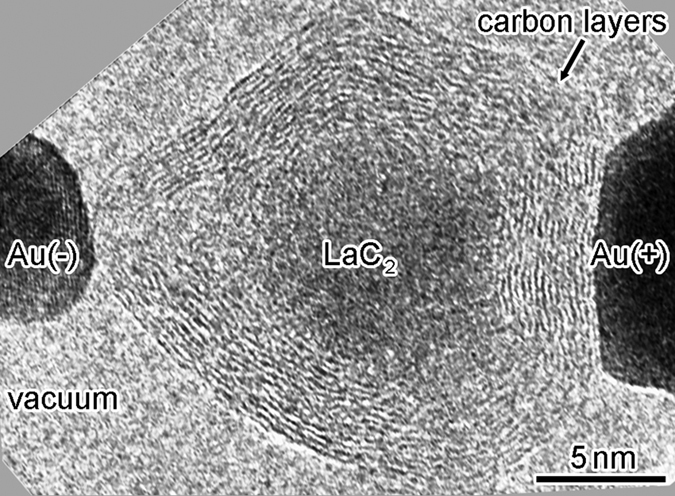
High-resolution TEM image of a Au/LaC_2_-encapsulating CNC/Au SNPJ. Au(+) and Au(−) show the positive and negative Au electrodes, respectively.

**Figure 6:**
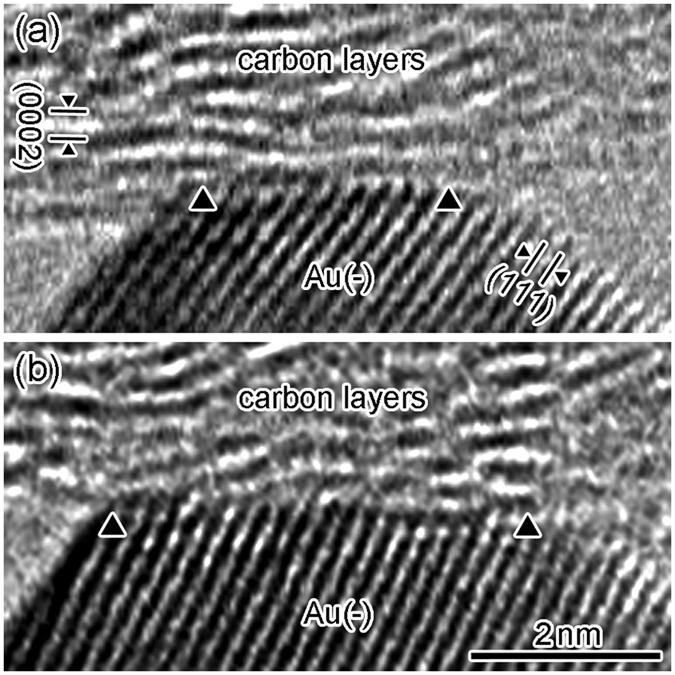
Enlarged images of the interface between the carbon layers and the negative Au electrode in the SNPJ shown in Fig. 5. The two large arrowheads indicate the edges of the contact region, i.e., the contact region is located between the two arrowheads.

Figure 5 shows a high-resolution TEM image of an SNPJ assembled using a LaC_2_-encapsulating CNC and two Au electrodes, similar to the case in Fig. 1(a). Figure 6 shows enlarged images of the interface between the carbon layers and the negative Au electrode in the SNPJ shown in Fig. 5. The sizes of the contact regions shown in Fig. 6(a,b) were controlled by manipulating the negative Au electrode with a fixed contact region between the CNC and the positive Au electrode (Supplementary Movie 2). The spacings of the lattice fringes observed in Fig. 6(a) are 0.34 and 0.24 nm, which correspond to (0002)_carbon layers_ and (111)_Au_, respectively. The widths of the interfaces between the carbon layers and the negative Au electrode shown in Fig. 6(a,b) are 3.4 nm and 5.0 nm, respectively. The total electrical resistance of the SNPJ (*R*_t_ Ω) is estimated using the following formula:


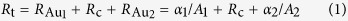


where *R*_Au1_ Ω and *R*_Au2_ Ω, *R*_c_ Ω, *α*_1_ Ω nm^2^ and *α*_2_ Ω nm^2^, and *A*_1_ nm^2^ and *A*_2_ nm^2^ are the contact electrical resistance between the CNC and the Au electrodes 1 and 2, the electrical resistance of the capsule part in the SNPJ, the contact electrical resistivity between the CNC and the Au electrodes 1 and 2, and the contact area between the CNC and the Au electrodes 1 and 2, respectively. The index number 1 or 2 is given by a case between the CNC and the positive or negative Au electrode. Here, we assumed that α_1_ is approximately equal to α_2_; thus, Equation (2) is given as follows.


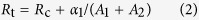


*R*_t_ and *A*_1_, *A*_2_ were 9.7 kΩ and 80.5 nm^2^, 9.1 nm^2^ (Fig. 6(a)) and 6.6 kΩ and 80.5 nm^2^, 19.4 nm^2^ (Fig. 6(b)), respectively. By substituting these values in Equation (2), *R*_c_ was obtained as 3.2 kΩ, and *R*_c_, *R*_Au1_, and *R*_Au2_ were 33%, 7%, and 60% of *R*_t_, respectively (Fig. 6(a)).

In this study, contact electrical resistivity is expressed using a unit of Ω∙nm^2 ^30,31. This is because we quoted the values of the contact electrical resistivity of graphene, which was expressed using Ω∙nm^2^ from previous studies^32^,^33^, as described in discussion, and developed the expression for the contact electrical resistance between the CNC and the Au electrode (Equation (1)).

## Discussion

As shown in Figs 4(b) and 6, *R*_t_ decreased as *A*_2_ increased. According to Equation (2), *R*_Au1_ and *R*_Au2_ decreased as *A*_1_ and *A*_2_ increased. The contact area between the CNC and the Au electrode of the SNPJ increased; thus, the contact electrical resistance decreased, resulting in decreased total electrical resistance of the SNPJ. In this study, the sum of *R*_Au1_ and *R*_Au2_ reached 67% of *R*_t_. Since the projected densities of states (PDOSs) of the π-orbitals of the graphene and the d-orbitals of the Au at near-Fermi energy overlap by a small amount, the electrical coupling of the metal with graphene becomes weak and the number of conduction channels decreases^34^. In addition, the transmission function near the Fermi energy mirrors the PDOS behaviors; therefore, the contact electrical resistance of the graphene and Au junctions becomes greater than that of the graphene and other metal junctions^34^.

**Figure 7:**
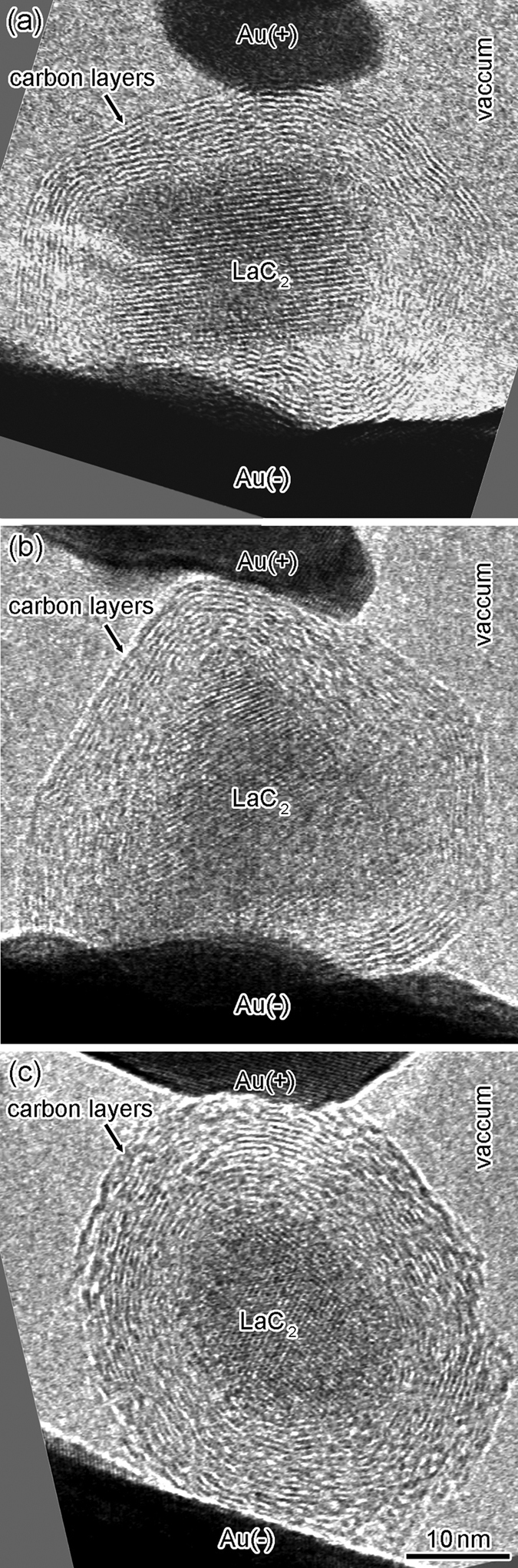
High-resolution TEM images of three Au/LaC_2_-encapsulating CNC/Au SNPJs. Au(+ ) and Au(−) show the positive and negative Au electrodes, respectively.

The I**–**V characteristics of the SNPJ presented in Fig. 1(a) show a linear relationship and no current region near 0 V, as shown in Fig. 2. These features are the same as those of graphene and the metal junctions^35^,^36^. Since the electrical conductivity of graphite along the in-plane direction is 10^4^ times greater than that along the c-axis direction^37^, we presume that the greatest portion of the current through the SNPJ flowed along the outermost carbon layer of the CNC. Here, we consider the electrical resistance of the graphene layers (*R*_g_) corresponding to the outermost layer of the CNC. *R*_g_ is expressed as follows^36^:


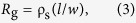


where ρ_s_ Ω per square, *l* nm, and *w* nm are the sheet resistance of graphene layers, the channel length, and the channel width, respectively. *l* was measured from the observed length between the positive and negative Au electrodes along the outermost carbon layer of the CNC. *w* is the perimeter of the contact area between the CNC and the negative Au electrode, and was calculated from the observed interface widths. The contact area between the CNC and the negative Au electrode was less than that of the positive electrode, as shown in Fig. 5, and the resistance of the graphene layers was dominated by the smaller contact area. Thus, we focused on the contact area between the CNC and the negative Au electrode using *w* in the contact region of the negative Au electrode to calculate *R*_g_. When ρ_s_ (1.8 Ω per square^38^), *l*, and *w* were substituted into Equation (3), *R*_g_ corresponding to the outermost layer of the CNC shown in Fig. 5 was approximately 3.0 kΩ. This value is similar to that of *R*_c_ in Equation (2). In addition, the relationships between *R*_c_ and *R*_g_ in the other three SNPJs assembled using LaC_2_-encapsulating CNCs shown in Fig. 7 were investigated. The results are shown in Table 1. We found that the *R*_*c*_ and *R*_g_ for each CNC show similar values.

There are wrinkles and kinks in the carbon layers of the CNC and it is considered that these contribute to current flows between the layers. Since it is difficult to evaluate the characteristics and quantity of these defects quantitatively, we used a simple model, in which the outermost layer of the CNC corresponded to graphene, and calculated the resistance of the CNC. As a result, we found that the measured resistance of the CNC agreed well with the calculated resistance using this simple model. This result suggests that although a certain amount of the winkles and kinks contributed to the current through between the layers, the contribution was substantially low in comparison with that of the current along the outermost layer.

In this study, we operated the electron microscope with an acceleration voltage of 200 kV and thus we also need to consider the introduction of radiation damages to the carbon layers and the effect on the conductance^39^. To reduce radiation damages as possible, we decreased the electron beam current to less than 4 A/cm^2^ and the total observation time to shorter than 1.5 min. During the image observation and the conductance measurement, no variation in the interlayer spacing of the carbon layers and no additional formation of radiation defects were observed although a certain amount of the radiation defects were inferred to be introduced. In this study, the major variation in electrical conductance of the SNPJ was observed when the contact area between the CNC and Au electrode was changed. Thus, although the introduction of a certain amount of the radiation damages also contributed to the variation in conductance, the contribution was minor and the total conductance was governed by the control of the contact areas.

The contact areas of the interfaces observed in this study were 30−90 nm^2^, which were near the lower limit for applying the Maxwell resistance approximation. As shown in the Fig. 4(b), since the inverse of the areas and the resistance have a linear relationship, it is considered that the approximation can be applied to the observed contact interfaces.

In summary, we investigated the structures and electrical properties of SNPJs assembled using LaC_2-_encapsulating CNCs. The electrical resistance of the CNC was estimated to be similar to that of the graphene layers corresponding to the outermost layer of the CNC, implying that the greatest portion of the current through the SNPJ flowed along the outermost carbon layer of the CNC. The contact electrical resistance decreased as the contact area between the CNC and the Au electrodes of the SNPJ increased; therefore, the total electrical resistance decreased. Based on this observation, the resistance and its origin of one isolated nanoparticle were evaluated. The contact electrical resistance was 67% of the total electrical resistance. This suggests that the electrical resistance of the SNPJ was significantly affected by the contact resistance. In this study, an electrical conduction mechanism of the SNPJ using the CNC was revealed. These results are expected to be applied to the design of SNPJs.

## Methods

The experimental method in this study was developed based on *in situ* TEM combined with piezomanipulation of nanotips and electric conductance measurements^29^. LaC_2_-encapsulating CNCs were synthesized via a gas evaporation method using dilanthanum trioxide (La_2_O_3_) and carbon^5^,^6^ evaporation sources. The synthesized LaC_2_-encapsulating CNCs were dispersed on the surfaces of a Au nanotip (the positive electrode). The nanotip, on which the CNCs were dispersed and a bare Au nanotip (the negative electrode) were mounted on the first and second sample holders, respectively and were inserted into a transmission electron microscope equipped with two goniometer stages and a sample piezodriving system. Inside the microscope, the negative Au electrode was manipulated using the piezodriving system at the picometer scale to pick up one CNC on the positive Au electrode, followed by sandwiching the CNC between the two electrodes. Bias voltage was then applied between the Au electrodes at room temperature at a vacuum of 1 × 10^−5^ Pa.

It is essential for this study to assemble controllable SNPJs. For this purpose, highly reproducible stable CNCs were required. The encapsulation of LaC_2_ into CNCs satisfies this requirement. Thus, to improve accuracy of the production and the control of SNPJs, we selected LaC_2_ as encapsulated nanoparticles.

## Additional Information

**How to cite this article**: Tezura, M. and Kizuka, T. Structures and electrical properties of single nanoparticle junctions assembled using LaC_2_-encapsulating carbon nanocapsules. *Sci. Rep.* **6**, 29708; doi: 10.1038/srep29708 (2016).

## Supplementary Material

Supplementary Information

Supplementary Movie 1

Supplementary Movie 2

## Figures and Tables

**Table 1:** Resistance of Au/LaC_2_-encapsulating CNC/Au SNPJs shown in Figs 5(a) and 7(a–c).

**SNPJ**	**encapsulating**	**CNC width (nm)**	**minor**	**the number of carbon**	**layers**	**resistance (kΩ)**
	**particles**	**major**			**R**_**c**_	**R**_**g**_
Fig. 5	LaC_2_	26	21	17	3.2 ± 0.4	3.0 ± 0.9
Fig. 7(a)	//	27	17	14	1.8 ± 0.4	2.4 ± 0.9
Fig. 7(b)	//	25	21	10	2.3 ± 0.4	2.4 ± 0.9
Fig. 7(c)	//	28	27	16	2.8 ± 0.4	2.4 ± 0.9

## References

[b1] Heidenreich R. D., Hess W. M. & Ban L. L. A test object and criteria for high resolution electron microscopy. J. Appl. Crystallogr. 1, 1–19 (1968).

[b2] Iijima S. Direct observation of the tetrahedral bonding in graphitized carbon black by high resolution electron microscopy. J. Cryst. Growth 50, 675–683 (1980).

[b3] Smith P. P. & Buseck P. R. Graphitic carbon in the allende meteorite: a microstructural study. Science 212, 322 (1981). 11536554 10.1126/science.11536554

[b4] Ugarte D. Curling and closure of graphitic networks under electron-beam irradiation. Nature 359, 707–709 (1992). 11536508 10.1038/359707a0

[b5] Tomita M., Saito Y. & Hayashi T. LaC_2_ encapsulated in graphite nano-particle. Jpn. J. Appl. Phys. 32, L280 (1993).

[b6] Rodney S. R., Donald C. L., Bryan C., Ripudaman M. & Shekhar S. Single crystal metals encapsulated in carbon nanoparticles. Science 259, 346 (1993). 17832348 10.1126/science.259.5093.346

[b7] Ugarte D. How to fill or empty a graphitic onion. Chem. Phys. Lett. 209, 99–103 (1993).

[b8] Majetich S. A., Artman J. O., McHenry M. E., Nuhfer N. T. & Staley S. W. Preparation and properties of carbon-coated magnetic nanocrystallites. Phys. Rev. B 48, 16845–16848 (1993). 10.1103/physrevb.48.16845 10008279

[b9] Brunsman E. M. . Magnetic properties of carbon-coated, ferromagnetic nanoparticles produced by a carbon-arc method. J. Appl. Phys. 75, 5882–5884 (1994).

[b10] Hihara T. . Magnetic properties of Iron in nanocapsules. Jpn. J. Appl. Phys. 33, L24 (1994).

[b11] El-Gendy A. A. . The synthesis of carbon coated Fe, Co and Ni nanoparticles and an examination of their magnetic properties. Carbon 47, 2821–2828 (2009).

[b12] Saito Y. Nanoparticles and filled nanocapsules. Carbon 33, 979–988 (1995).

[b13] Saito Y. & Matsumoto T. Hollow and filled rectangular parallelopiped carbon nanocapsules catalyzed by calcium and strontium. J. Cryst. Growth 187, 402–409 (1998).

[b14] Reed M. A., Zhou C., Muller C. J., Burgin T. P. & Tour J. M. Conductance of a molecular junction. Science 278, 252–254 (1997).

[b15] Park H. . Nanomechanical oscillations in a single-C_60_ transistor. Nature 407, 57–60 (2000). 10993069 10.1038/35024031

[b16] Taylor J., Guo H. & Wang J. *Ab initio* modeling of quantum transport properties of molecular electronic devices. Phys. Rev. B 63, 245407 (2001).

[b17] LeRoy B. J., Lemay S. G., Kong J. & Dekker C. Electrical generation and absorption of phonons in carbon nanotubes. Nature 432, 371–374 (2004). 15549099 10.1038/nature03046

[b18] Yaish Y. . Electrical nanoprobing of semiconducting carbon nanotubes using an atomic force microscope. Phys. Rev. Lett. 92, 046401 (2004). 14995390 10.1103/PhysRevLett.92.046401

[b19] Dadosh T. . Measurement of the conductance of single conjugated molecules. Nature 436, 677–680 (2005). 16079841 10.1038/nature03898

[b20] LeRoy B. J., Kong J., Pahilwani V. K., Dekker C. & Lemay S. G. Three-terminal scanning tunneling spectroscopy of suspended carbon nanotubes. Phys. Rev. B 72, 075413 (2005).

[b21] Tao N. J. Electron transport in molecular junctions. Nat. Nanotechnol. 1, 173–181 (2006). 18654182 10.1038/nnano.2006.130

[b22] Asaka K., Kato R., Yoshizaki R., Miyazawa K. & Kizuka T. Conductance of carbon nanocapsule junctions. Phys. Rev. B 76, 113404 (2007).

[b23] Sun C. Q. Thermo-mechanical behavior of low-dimensional systems: The local bond average approach. Prog. Mater. Sci. 54, 179–307 (2009).

[b24] Huang T. . A molecular switch based on current-driven rotation of an encapsulated cluster within a fullerene cage. Nano Lett. 11, 5327–5332 (2011). 22081996 10.1021/nl2028409

[b25] Néel N., Kröger J. & Berndt R. Two-level conductance fluctuations of a single-molecule junction. Nano Lett. 11, 3593–3596 (2011). 21854026 10.1021/nl201327c

[b26] Aradhya S. V. & Venkataraman L. Single-molecule junctions beyond electronic transport. Nat. Nanotechnol. 8, 399–410 (2013). 23736215 10.1038/nnano.2013.91

[b27] Evangeli C. . Engineering the thermopower of C_60_ molecular junctions. Nano Lett. 13 (2013). 10.1021/nl400579g 23544957

[b28] Matsuura D. & Kizuka T. Electrical conductivity of single molecular junctions assembled from Co- and Co_3_C-encapsulating carbon nanocapsules. J. Nanosci. Nanotechnol. 14, 2441–2445 (2014). 24745244 10.1166/jnn.2014.8506

[b29] Kizuka T. Atomic configuration and mechanical and electrical properties of stable gold wires of single-atom width. Phys. Rev. B 77, 155401 (2008).

[b30] Yu A. Y. C. Electron tunneling and contact resistance of metal-silicon contact barriers. Solid·State Electron. 13, 239–247 (1970).

[b31] Lin M. E. . Low resistance ohmic contacts on wide band-gap GaN. Appl. Phys. Lett. 64, 1003–1005 (1994).

[b32] Venugopal A., Colombo L. & Vogel E. M. Contact resistance in few and multilayer graphene devices. Appl. Phys. Lett. 96, 013512 (2010).

[b33] Moon J. S. . Ultra-low resistance ohmic contacts in graphene field effect transistors. Appl. Phys. Lett. 100, 203512 (2012).

[b34] Matsuda Y., Deng W.-Q. & Goddard W. A. Contact resistance properties between nanotubes and various metals from quantum mechanics. J. Phys. Chem. C 111, 11113–11116 (2007).

[b35] Castro Neto A. H., Guinea F., Peres N. M. R., Novoselov K. S. & Geim A. K. The electronic properties of graphene. Rev. Mod. Phys. 81, 109–162 (2009).

[b36] Peng S.-A. . The sheet resistance of graphene under contact and its effect on the derived specific contact resistivity. Carbon 82, 500–505 (2015).

[b37] Primak W. & Fuchs L. H. Electrical conductivities of natural graphite crystals. Phys. Rev. 95, 22–30 (1954).

[b38] Wang X., Zhi L. & Müllen K. Transparent, conductive graphene electrodes for dye-sensitized solar cells. Nano Lett. 8, 323–327 (2008). 18069877 10.1021/nl072838r

[b39] Smith B. W. & Luzzi D. E. Electron irradiation effects in single wall carbon nanotubes. J. Appl. Phys. 90, 3509–3515 (2001).

