# Supplementary material for: Structures and electrical properties of single nanoparticle junctions assembled using LaC2-encapsulating carbon nanocapsules
Source: Sci Rep. 2016 Jul 14;6:29708. doi: 10.1038/srep29708 (PMC4944215; doi:10.1038/srep29708)
Supplement: Supplementary Information [file srep29708-s1.pdf]

Supplementary Movies

Title: **Structures and electrical properties of single nanoparticle junctions assembled using LaC_2-encapsulated carbon nanocapsules**

Authors: Manabu Tezura and Tokushi Kizuka

Author affiliation: Division of Materials Science, Faculty of Pure and Applied Sciences, University of Tsukuba, Tsukuba, Ibaraki 305-8573, Japan

Movies

Movie 1 | Movie of *in situ* high-resolution TEM of the formation of a Au/LaC_2-encapsulated CNC/Au single nanoparticle junction by piezomanipulation, corresponding to Fig. 1 and successive interfacial structural control.

Movie 2 | Movie of the control process of size of a contact interface between the carbon layers and Au electrode using the piezo-driving system, similar to the process shown Fig. 6 (the observed material corresponds to Fig. 7(b)).
